# Draft genome sequence data on *Bacillus safensis* FB03 isolated from the rhizosphere soil of leguminous plant in Bangladesh

**DOI:** 10.1016/j.dib.2025.111527

**Published:** 2025-03-27

**Authors:** Farhana Boby, Dr. Md Nurul Huda Bhuiyan, Dr. Md. Salim Khan, Md. Mashud Parvez

**Affiliations:** a BCSIR Laboratories, Dhaka, Bangladesh Council of Scientific and Industrial Research (BCSIR), Dhaka 1205, Bangladesh; b BCSIR Rajshahi Laboratories, Bangladesh Council of Scientific and Industrial Research (BCSIR), Rajshahi 6206, Bangladesh

**Keywords:** *Bacillus safensis*' FB03, Biotechnology, Genome annotation, Biosynthetic Gene cluster, Pangenome

## Abstract

With the aim of investigating the biotechnological potential of *Bacillus safensis*' FB03, isolated from the rhizosphere soil of Bahrind region of Bangladesh, the current work focused on its complete genomic analysis and phenotypic description. The size of the genome of the isolate was 3.6 Mb with 41.59 % GC content. Genome annotation revealed the presence of many genes related to siderophore production, enzyme degradation, UV and stress tolerance. Six biosynthesis gene clusters for bacillibacin, bacilysin, bottromycin, Schizokinen, fengycin, and lychensin were identified through genome mining. Significantly, FB03 was found to contain only two acquired antimicrobial resistance genes and was anticipated to be non-pathogenic to humans. The openness of the *Bacillus safensis* pan-genome was demonstrated by the pan-genome analysis. According to this research, *Bacillus safensis* FB03 may be a good fit for a variety of biotechnological applications.

## Graphical abstract

**Figure:**
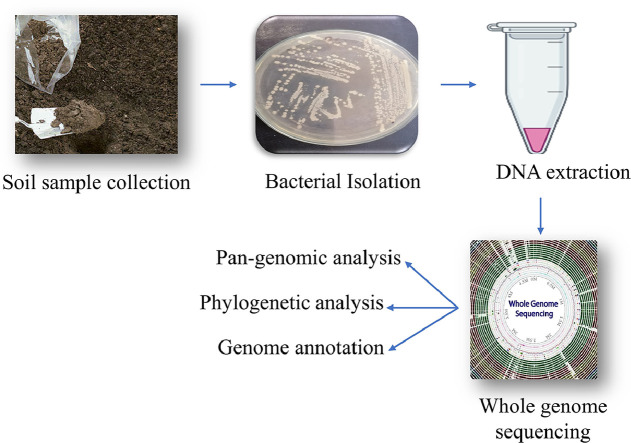

Specification Table

**Table:** 

Subject	Microbiology • Applied Microbiology
Specific subject area	Omics: Genomics
Type of Data	Raw, filtered, table, figure, analyzed, deposited.
Data Collection	The strain was isolated from the rhizosphere soil of a leguminous plant grown in the Bahrind region (N 24°22ˊ1˝, E 88°39ˊ15˝), Rajshahi division, Bangladesh. Genomic DNA was extracted and sequenced using Illumina Miseq technology. Quality-filtered trimmed reads were assembled using SPAdes genome assembler V3.15.5 after adapter trimming by Trimmomatic version 0.36. A genomic map was constructed with the help of Proksee. Type (Strain) Genome Server (TYGS) and MEGA v11 were employed for phylogenetic analysis. Pathogenicity and virulence were assesses using PathogenFinder, VFDB, CARD. AntiMASH and BAGEL4 were used for genome annotation, while IPGA v1.09 was used to performed Pangenomic analysis.
Data Source Location	Institution: Bangladesh Council of Scientific and Industrial Research (BCSIR), Rajshahi, Bangladesh.
Data Accessibility	Repository name: NCBI (National Center for Biotechnology Information) Data Identification number: Bioproject: PRJNA1161822 Biosample: SAMN43792388 Direct URL to data: https://www.ncbi.nlm.nih.gov/biosample/?term=SAMN43792388
Related research article	No

## 1 Value of Data

• The draft genome sequence of *B. safensis* FB03 would be valuable for analyzing bacterial ecology and taxonomy, specifically in the context of systematic identification and dispersion.
• The content of this article may be advantageous for researchers studying the genetic structure and production of secondary metabolites, particularly the non-ribosomal peptides which have therapeutic and other biotechnological applications.
• To utilize *B. safensis* as a plant growth promoter in the halophilic environment, this genomic data can serve as a useful basis for cloning and expression of salt tolerant genes.
• The sequence data may provide valuable insight for research on comparative genomic analysis among different subspecies in the environment.

## 2 Background

*Bacillus safensis* is a Gram-positive, aerobic, spore-forming bacterium with high tolerance to salt, heavy metal, UV and gamma radiation [1,2]. Additionally, it serves as a plant growth promoter, bio-control agent and bio-remediating organism [3,4]. Its ability to synthesize various industrially important enzymes and other secondary metabolites, makes it advantageous for a variety of biotechnology applications [5,6]. To date, no *Bacillus safensis* has been reported from Bangladesh.

Therefore, the present study aimed to isolate and identify *Bacillus safensis* FB03 from the rhizosphere soil of the Bahrind region of Bangladesh. The whole genome sequence analysis was conducted to assess its phylogenetic relationship with closely related species, biotechnological applications and survival under extreme conditions. Besides, to evaluate its safety as an industrially important isolate, virulence and resistance profile were also studied.

## 3 Data Description

### 3.1 Genomic features and phylogenetic analysis of *Bacillus safensis* FB03

The assembled genome of FB03 was 3.5 Mb and found to be distributed into 39 contigs, with 41.59 % GC content (Table 1). The genome is complete with a low contamination (0.2 %) and has an N50 value of 937,621 and L50 of 2. Genome annotation by PROKKA predicted 3677 protein-coding sequences, 5 rRNA, and 45 tRNA in the draft assembly.

**Table 1:** Characteristics of *B. safensis* FB03 assembled genome.

Characteristics	Terms	
Taxonomy	Firmicute> Bacilli> Bacilales>Bacilaceae>Bacillus>Bacillus safensis	
Genomic statistics	Size	3,614,609 bp
	Number of contigs	39
	GC content (%)	41.59
	Contig N50 value	937,621
	Contig L50 value	2
Genomic feature	CDC	3677
	tRNA	45
	rRNA	5
Genome quality	Completeness	100 %
	Contamination	0.3 %
	Overall remarks	Good
Genome availability	Bioproject	PRJNA1161822
	Biosample	SAMN43792388
	GenBank accession	JHHILD000000000

**Fig. 1:**
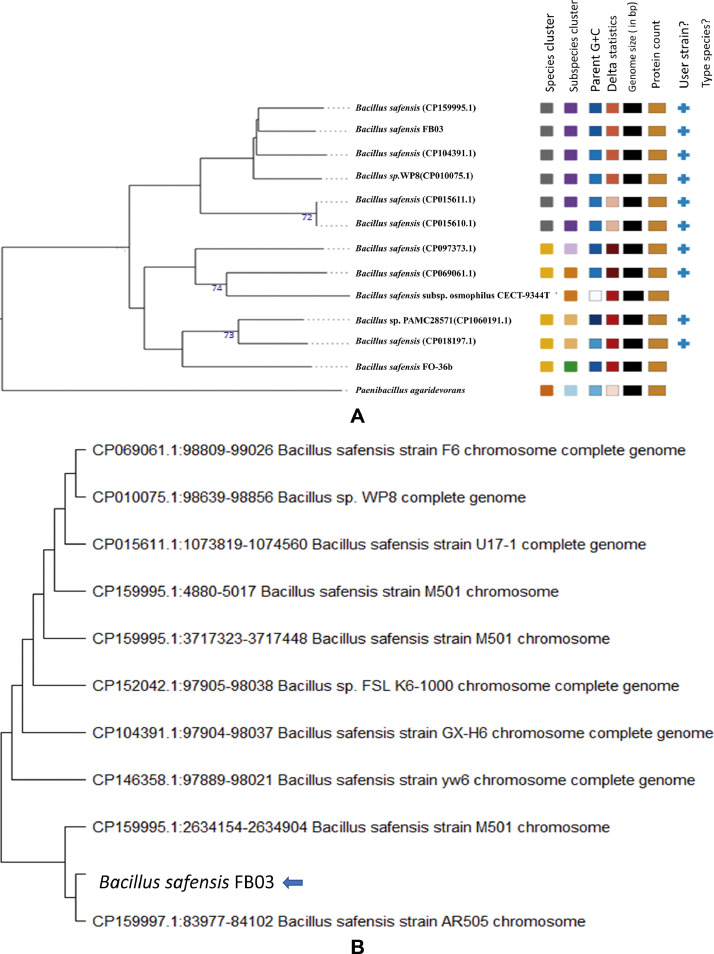
**A:** Phylogenetic analysis of *B. safensis* FB03 based on Genome Blast Distance Phylogeny (GBDP) approach in TYGS **B:** Phylogenetic analysis of *B. safensis* FB03 based on codon tree in MEGA v11.

The bacterium was identified as *Bacillus safensis* by whole genome taxonomy analysis using TYGS and GTDB. While *B. safensis* M501 (CP159995.1) was shown to be FB03’s closest neighbor in a whole genome phylogenetic tree based on GBDP (Fig. 1A). Codon tree-based phylogenetic analysis indicated that FB03 was similar to *B. safensis* strain AR505GX (CP159997.1) (Fig. 1B).

### 3.2 Antibiotic resistance and virulence genes prediction

**Fig. 2:**
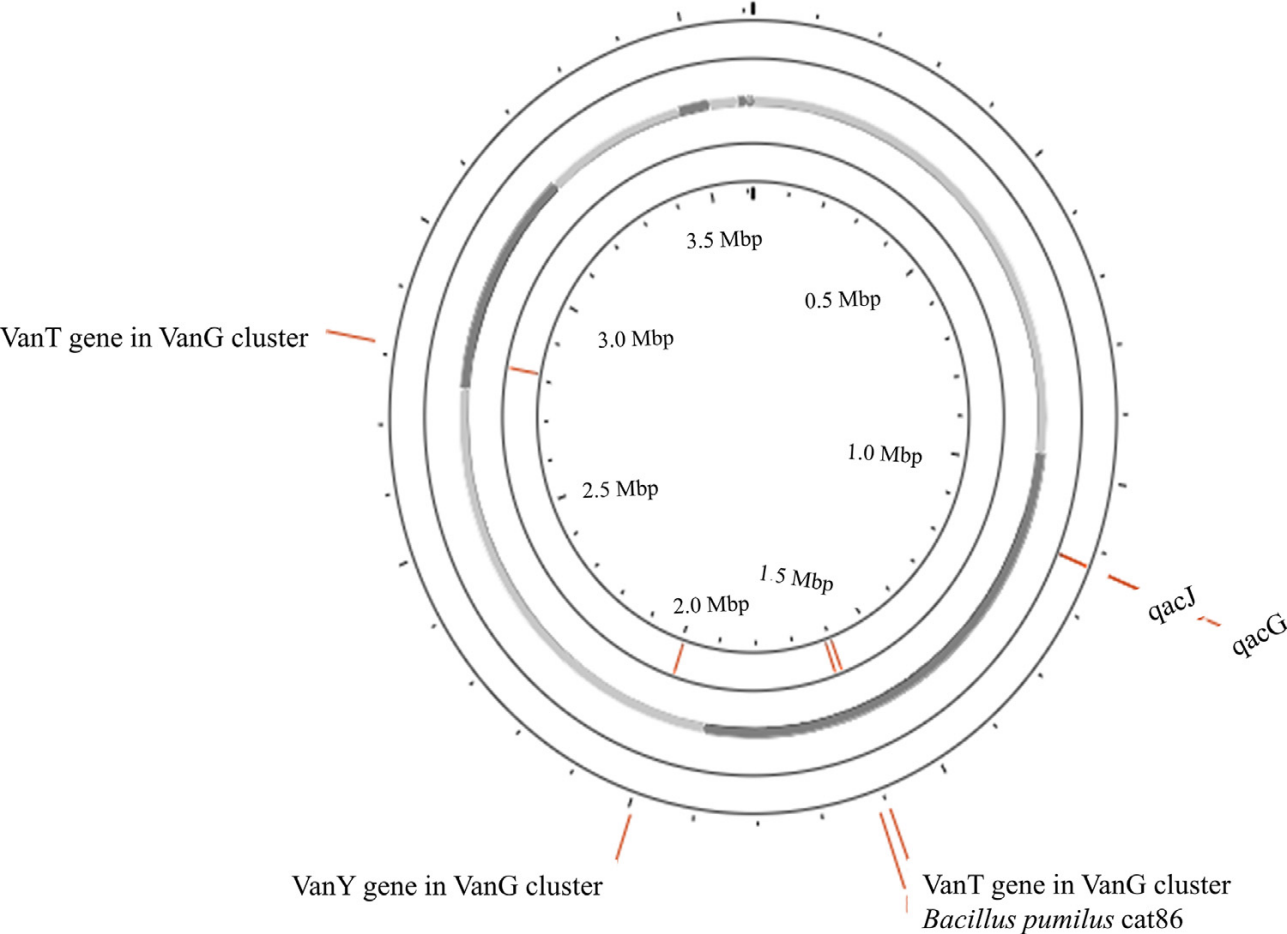
Resistant genes identified by CARD.

According to pathogen Finder, *B.safensis* FB03 did not exhibit any pathogenicity towards humans. Although, Virulence Factor Database (VFDB) analysis resulted in the detection of 2 virulence factors tufa (elongation factor) and clpC (endopeptidase), they are not virulent like hemolysin, cytotoxin, enterotoxin. A search for the antimicrobial resistant genes in FB03 genome against the CARD database identified the presence of vancomycin resistant gene cluster vanG with vanT, vanY genes. Additionally, 2 genes, qacJ and qacG, associated with resistance to disinfectants, were also identified (Fig. 2).

### 3.3 Genome annotation and Biosynthetic gene cluster (BGC) identification

**Fig. 3:**
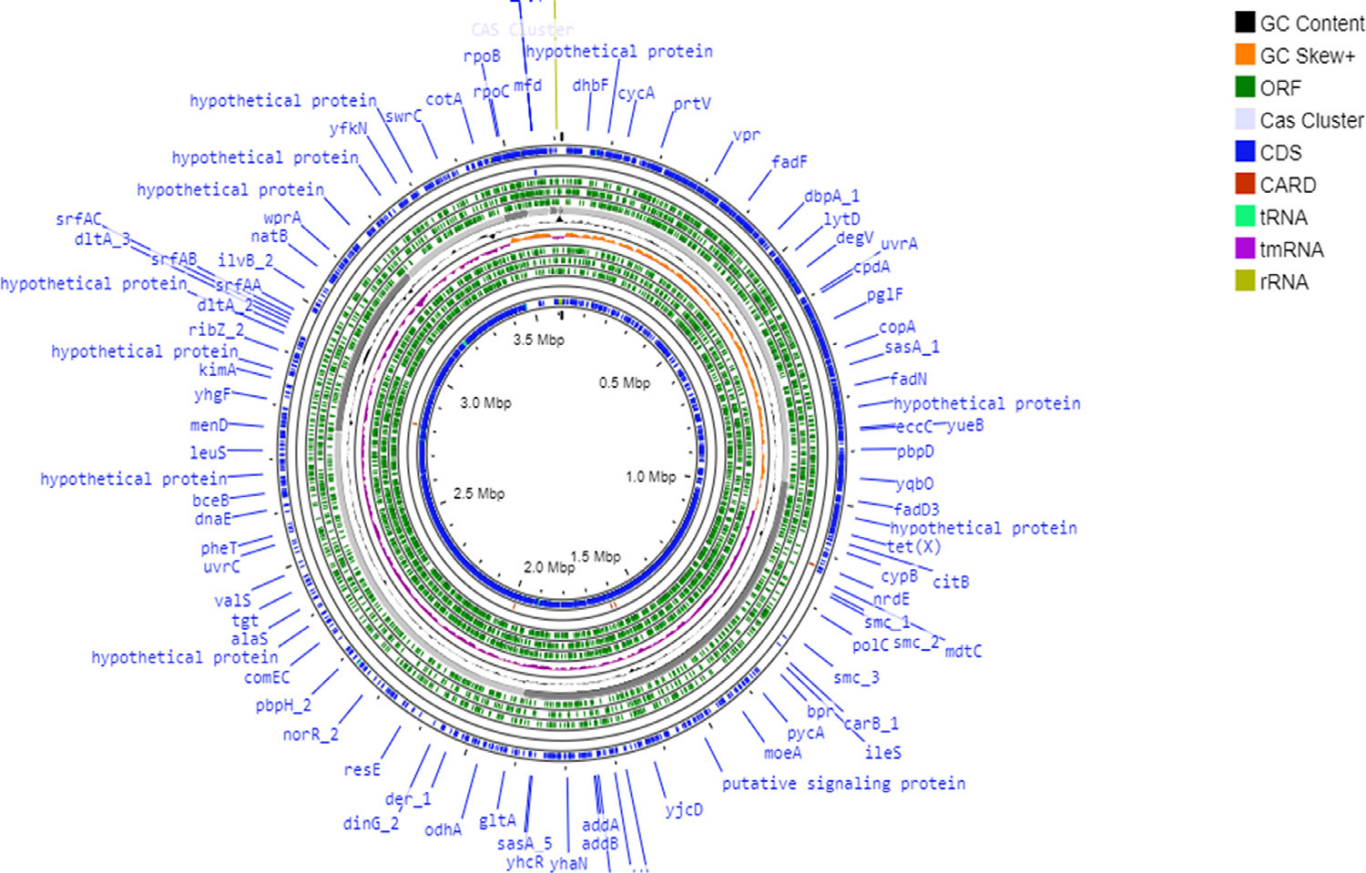
Circular genome map of *B.safensis* FB03 with different annotation characteristics.

**Fig. 4:**
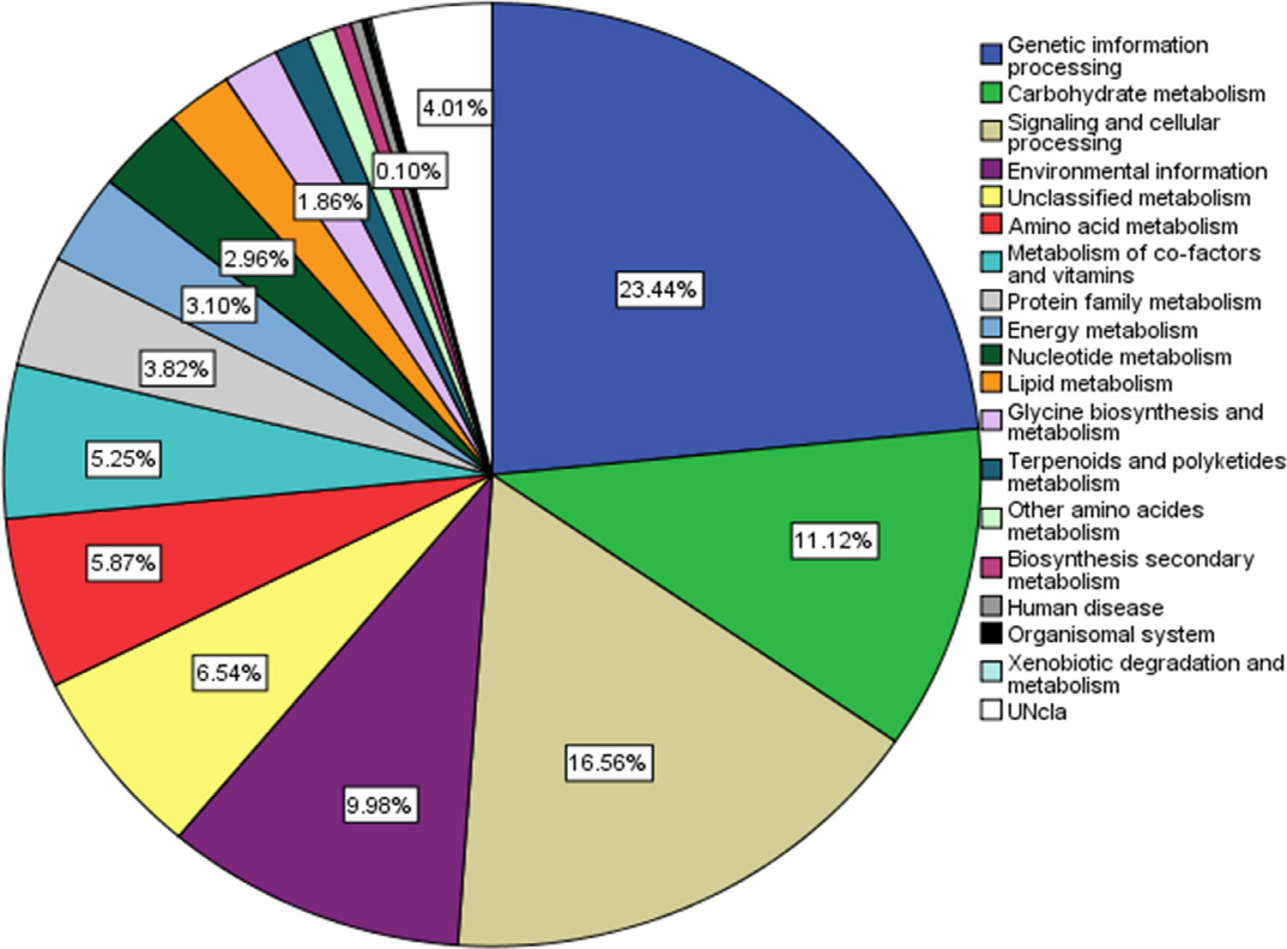
Functional annotation of *B.safensis* FB03: KEGG protein annotation.

**Fig. 5:**
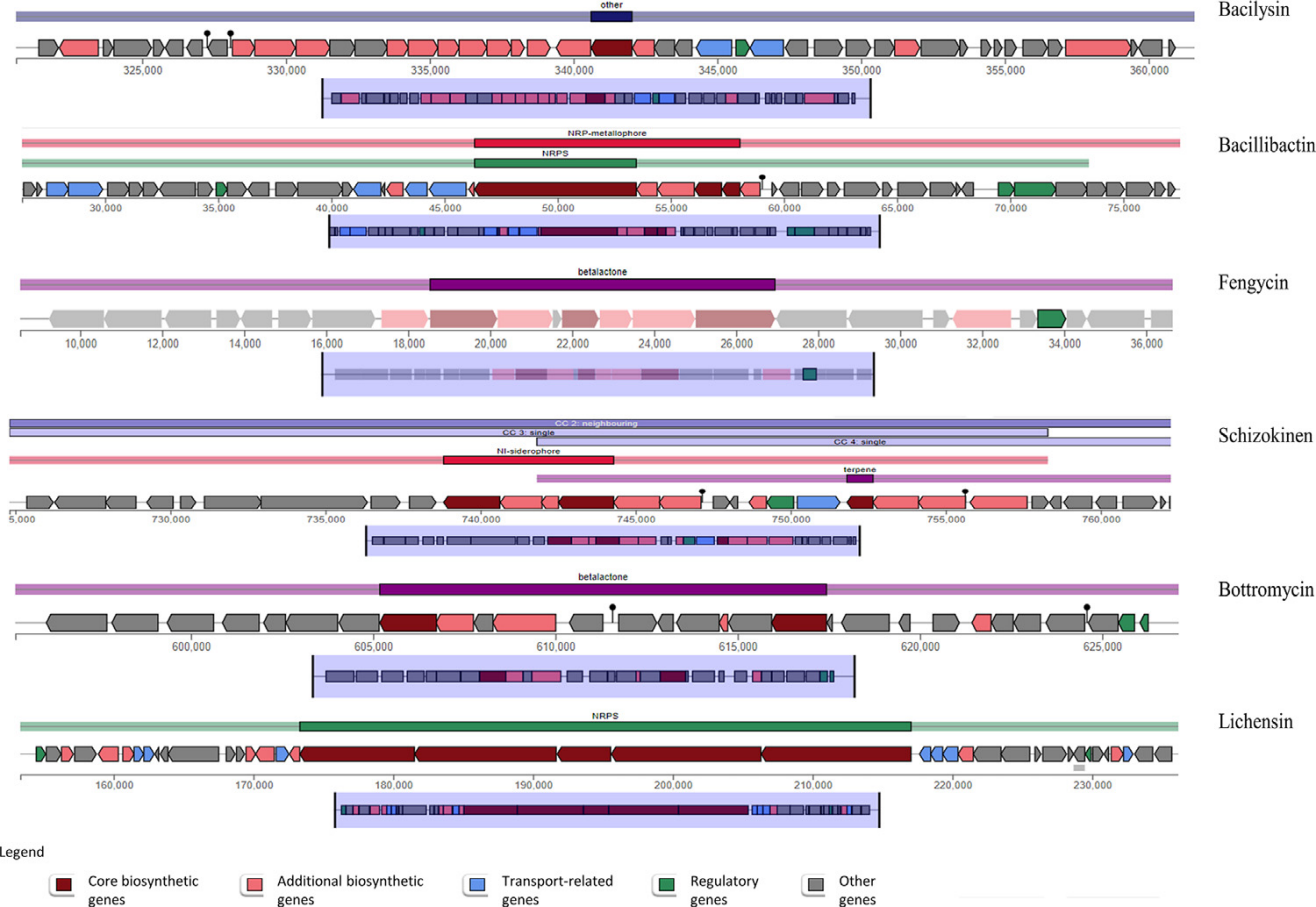
Schematic diagram of secondary metabolites biosynthetic gene clusters in *B.safensis* FB03.

Genome annotation using PROKKA predicted 3677 protein -coding sequences, 5 rRNA, and 45 tRNA in the draft assembly (Fig. 3). Additionally, FB03 harbored 12 genes associated with the synthesis of secondary metabolites, 2 involved in degradation of xenobiotic compound like pesticides, 1 involved in salt stress tolerance and 1 for UV radiation. Furthermore, KEGG protein annotation of 3771 queries resulted in 2166 entities connected to numerous pathways. The resulting data identified that majority of the pathways are associated with genetic information processing, signaling and cellular information processing, and metabolisms (Fig. 4). The FB03 genome has been found to harbor six gene clusters (BGC) involved in biosynthesis, which code for bacillibactin, bacilysin, fengycin, schizokinen, bottromycin, and lichenysin. These BGC consists of core and additional biosynthetic genes, regulatory, transporter, and other genes (Fig. 5). BAGEL4 identified five gene clusters of interest related to the synthesis of secondary metabolites, including sactipeptide, pumilarin, UviB, comX, and lanthipeptide class IV (Table 2).

**Table 2:** Secondary metabolites synthesizing clusters identified by BAGEL4.

Area of interest	Position		Class
	Start	End	
NODE_5_length_280714_cov_15909158.0.	226,775	246,775	Lanthipeptide class_IV
NODE_5_length_280714_cov_15909158.0.	67,172	87,172	Sactipeptides
NODE_3_length_856257_cov_15679349.2.	453,530	473,851	Pumilarin
NODE_1_length_970088_cov_16222310.6.	865,550	885,718	ComX1
NODE_1_length_970088_cov_16222310.6.	949,700	969,889	UviB

### 3.4 Pan-genome analysis

**Fig. 6:**
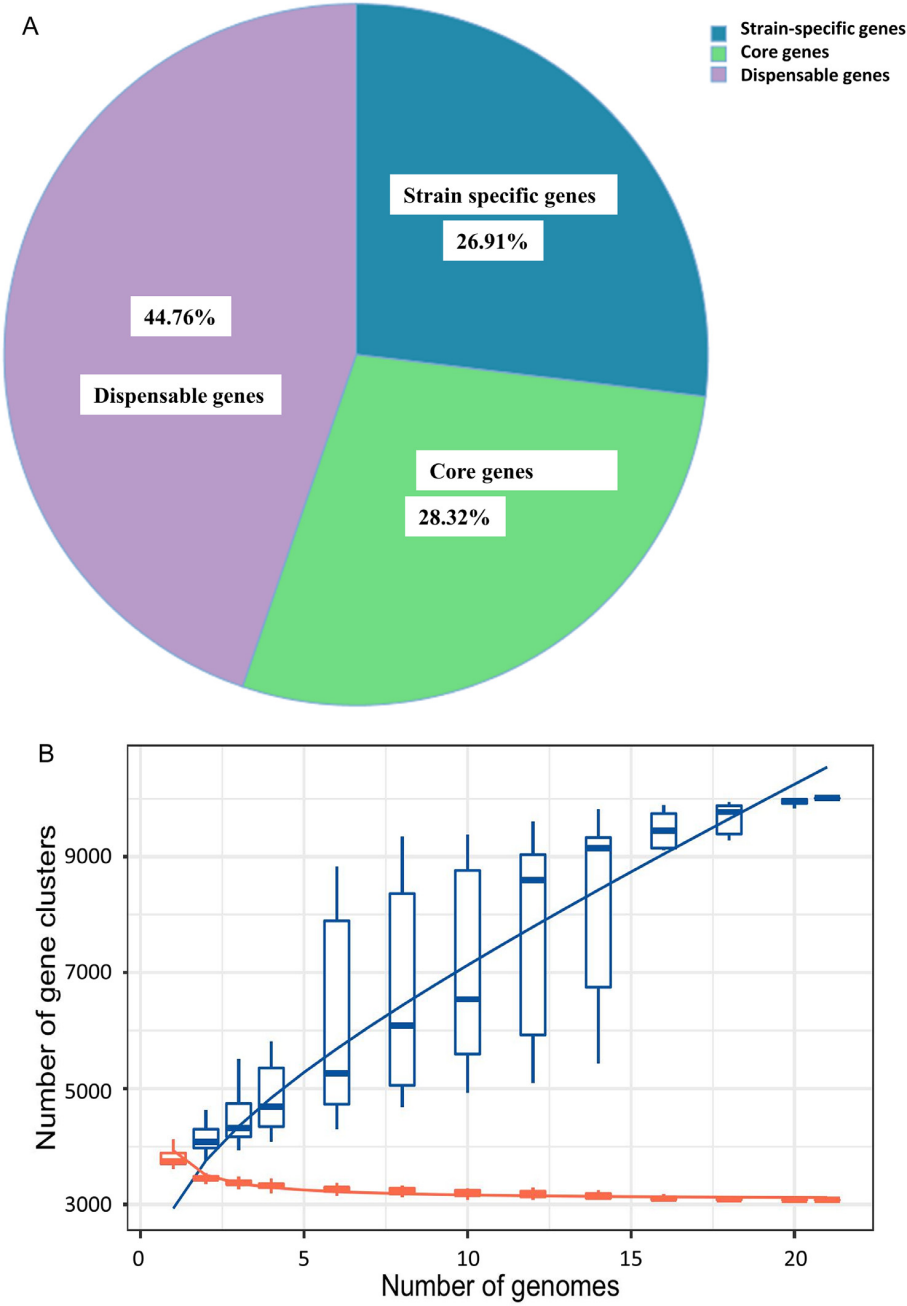

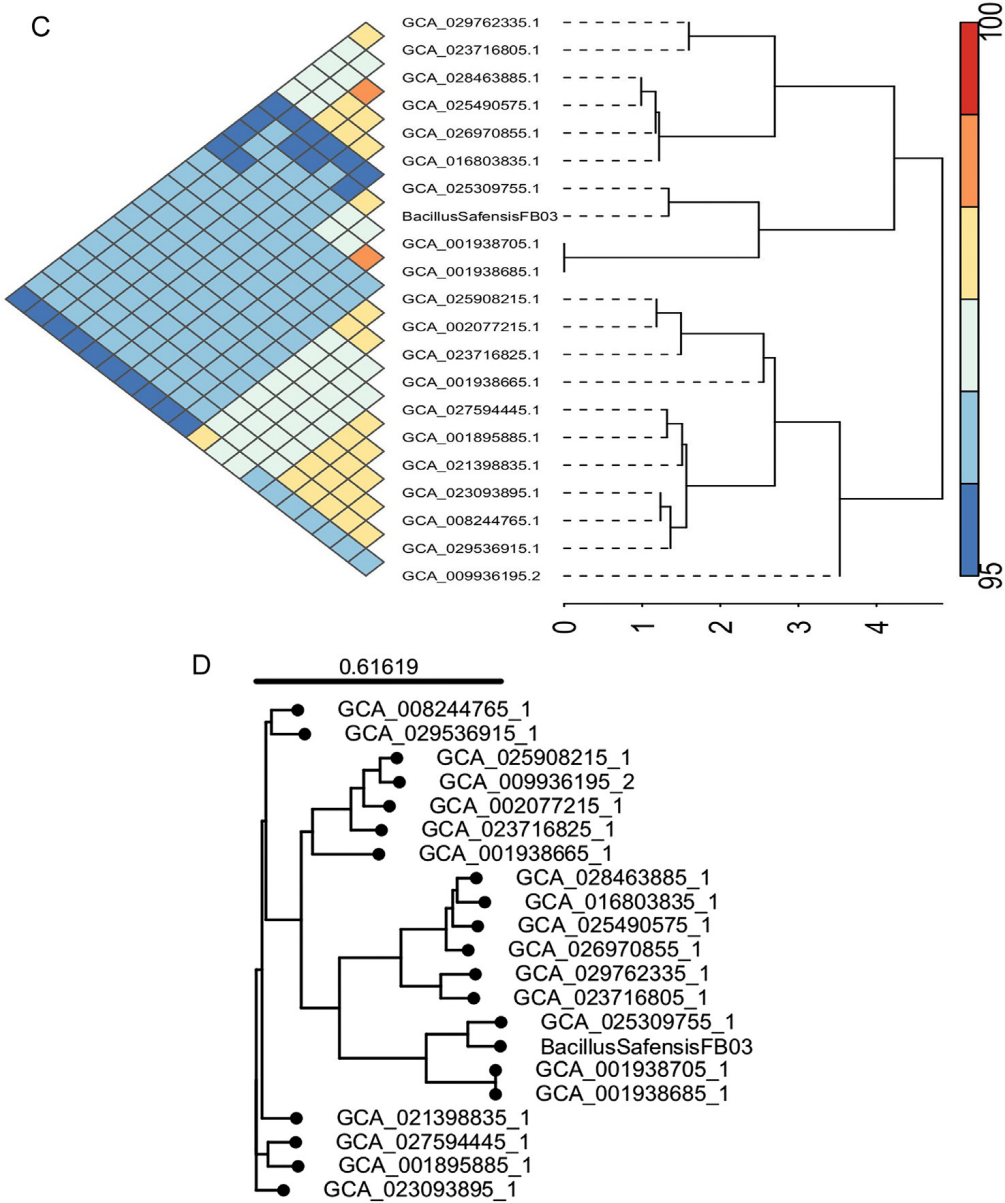
**A**: Pan-genome analysis of B. *safensis strains*: Distribution of core, accessory genes and strain specific genes **B**: Pan-genome analysis of B. *safensis strains*: Pan-genome size rarefaction curve **C**: Pan-genome analysis of B. *safensis strains*: Comparison of ANI values between strains **D**: Pan-genome analysis of B. *safensis strains*: Pan-genome phylogenetic tree.

Roary-based pan-genome analysis identified 3619 clusters, including 974 strain-specific genes (26.91 %), 1025 core genes (28.32 %) and 1620 dispensable genes (44.76 %) (Fig. 6A). The core-pan rarefaction curve demonstrated that the inclusion of additional strains led to a progressive increase in the number of pan genome genes, while the core genome showed the reverse trend (Fig. 6B). *B. safensis* FB03 pan-genome was found to be opened, as no discernible plateau was observed in the core/pan-genome ratio. ANI-based pan-genome analysis revealed that FB03 shared a close phylogenetic relationship with other examined isolates which had ANI values of 95 % or more (Fig. 6C). Furthermore, phylogenetic tree, generated by hierarchical clustering from binary matrix (presence/absence) of accessory genes suggested that FB03 clustered in the same clade with strains accession number GCA_025309755.1, GCA_001938705.1, GCA_001938685.1 (Fig. 6D).

## 4 Experimental Design, Materials and Methods

The Bahrind soil of the Rajshahi region was selected as it is endemic to the northern part of Bangladesh. Bacteria were isolated from the rhizosphere soil of a maize crop at the BCSIR Rajshahi laboratory, Bangladesh. Using standard dilution method, the isolate was obtained on a nutrient agar plate when incubated at 37 °C for 24 h. To verify purity and study distinct colony characteristics, the resulting discrete colony was sub-cultured for several times.

### 4.1 Genome sequencing and assembly

Bacterial DNA was sequenced at Invent technologies Bangladesh Ltd. using Illumina Miseq technology for paired end-whole genome sequencing with 30X sequence coverage. FastQC was used to examine the raw sequence quality characteristics. Quality-filtered trimmed reads were assembled using SPAdes genome assembler V 3.15.5 after adapter trimming by Trimmomatic version 0.36 applying isolates parameters for high coverage isolate and multi-cell illumina data [7]. Finally, QUAST v5.2.0 was used to assess the quality of assembled genome [8]. After removing contigs shorter than 200 bp, the draft genome was uploaded to NCBI GenBank.

### 4.2 Phylogenetic analysis and functional annotation

For whole genome-based taxonomic identification and categorization of bacteria, the assembled genome was uploaded to the Type (Strain) Genome Server (TYGS) [9] and MEGA v11 [10]. TYGS constructed a whole-genome phylogenetic tree using the Genome Blast Distance Phylogeny (GBDP) approach whereas, MEGA utilized the neighborhood joining method. Genome annotation and target gene identification were performed using Proksee with default parameters [11]. The KEGG Automatic Annotation Server was used to study the metabolic pathway of assembled genome.

### 4.3 Characterization of virulence and other properties

Using CARD with minimum identity and coverage of 80 %, contigs of the assembled genome were tested to identify antimicrobial resistance genes. To identify virulence factor, and pathogenicity, the Virulence Factor Database (VFDB), Pathogen Finder V1.1 were utilized. AntiMASH (bacterial version) and BAGEL4 were employed in the genome mining process to find out the secondary metabolite biosynthesis gene clusters and bacteriocins.

### 4.4 Pan-genomic analysis

The NCBI GenBank database was used to obtain the genome sequences of twenty distinct strains of *Bacillus safensis* (Supplementary material). Using IPGA v1.09, the sequences were subjected to pan-genome analysis by Roary for genome clustering and analytic processes.

## Limitation

Not applicable

## Ethics Statement

The authors have read and followed the ethical requirements for publication in Data in Brief and confirming that the current work does not involve human subjects, animal experiments, or any data collected from social media platforms.

## Data availability statement

All data generated or analyzed during this study are included in this published article and microbe sample from *Bacillus safensis* FB03*.*

## CRediT Authorship Contribution Statement

**Farhana Boby:** Writing- original draft, methodology, Formal analysis, Data curation, **Nurul Huda:** Writing- review and editing, Supervision, Conceptualization. **Salim Khan:** Investigation, Funding acquisition, **Mashud Parvez:** Methodology, Formal analysis.

## Funding

The financial supported for this work was provided by Bangladesh Council of Scientific and Industrial Research (BCSIR), Dhaka, Bangladesh.

## Data Availability

National Center for Biotechnology InformationWhole genome data of Bacillus safensis FB03 (Original data).
